# Association of Cyclin-Dependent Kinases 4 and 6 Inhibitors With Survival in Patients With Hormone Receptor–Positive Metastatic Breast Cancer: A Systematic Review and Meta-analysis

**DOI:** 10.1001/jamanetworkopen.2020.20312

**Published:** 2020-10-13

**Authors:** Jinming Li, Xingfa Huo, Fuxing Zhao, Dengfeng Ren, Raees Ahmad, Xinyue Yuan, Feng Du, Jiuda Zhao

**Affiliations:** 1 Breast Disease Diagnosis and Treatment Center of Affiliated Hospital of Qinghai University & Affiliated Cancer Hospital of Qinghai University, Xining, China; 2 Key Laboratory of Carcinogenesis and Translational Research (Ministry of Education/Beijing), The VIPII Gastrointestinal Cancer Division of the Medical Department, Peking University Cancer Hospital and Institute, Beijing, China

## Abstract

**Importance:**

One of the most recent treatment regimens used for hormone receptor (HR)–positive, *ERBB2* (formerly *HER2*)–negative metastatic breast cancer is treatment with the cyclin-dependent kinases 4 and 6 (CDK4/6) inhibitors and endocrine therapy (ET).

**Objective:**

To assess overall survival (OS), progression-free survival (PFS), objective response rate, and adverse events, especially grades 3 and 4 adverse events, among patients with HR-positive, *ERBB2*-negative metastatic breast cancer who were treated with CDK4/6 inhibitors plus ET vs ET alone.

**Data Sources:**

A systematic search of PubMed, Embase, the main oncology conference of the European Society of Medical Oncology, and the American Society of Clinical Oncology and the San Antonio Breast Cancer Symposium databases for randomized clinical trials of CDK4/6 inhibitors plus ET vs ET for HR-positive, *ERBB2*-negative metastatic breast cancer. Searches were performed up to March 30, 2020.

**Study Selection:**

A total of 472 records were assessed in PubMed and Embase by 2 authors, including studies, international meeting reports, and reviews. Inclusion criteria were English-language phase 2 or 3 randomized clinical trials of HR-positive, *ERBB2*-negative metastatic breast cancer, with patients randomly assigned to receive CDK4/6 inhibitors plus ET or ET alone, and having OS or PFS outcomes. The exclusion criteria were phase 1 trials, retrospective studies, or studies without survival outcomes. Excluding the references, 16 articles were relevant. After excluding studies based on exclusion criteria, 9 studies were considered eligible for this meta-analysis.

**Data Extraction and Synthesis:**

Two researchers independently extracted data and assessed potential bias. Data assessment followed the Preferred Reporting Items for Systematic Reviews and Meta-analyses reporting guideline. The results were pooled using a fixed-effect model.

**Main Outcomes and Measures:**

Study heterogeneity was assessed using the *I^2^* statistic. Hazard ratios (HRs) and 95% CIs were used to evaluate PFS, OS, and subgroup analyses. Overall response and 95% CIs were used to evaluate the objective response rate and grade 3 or 4 adverse events. The primary outcome was OS.

**Results:**

In total, 9 studies that included a total of 5043 patients with metastatic breast cancer were assessed in this meta-analysis. Overall, the addition of CDK4/6 inhibitors to ET was associated with a statistically significant benefit to OS (HR, 1.33; 95% CI, 1.19-1.48; *P* < .001). Compared with ET alone, treatment with CDK4/6 inhibitors plus ET was associated with improved OS for the following subgroups: first-line therapy (HR, 1.35; 95% CI, 1.18-1.54; *P* < .001), second-line therapy (HR, 1.30; 95% CI, 1.09-1.54; *P* < .001), premenopausal women (HR, 1.32; 95% CI, 1.04-1.66; *P* < .001), postmenopausal women (HR, 1.34; 95% CI, 1.18-1.52; *P* < .001), visceral metastasis (HR, 1.31; 95% CI, 1.12-1.53; *P* < .001), bone-only metastasis (HR, 1.22; 95% CI, 0.88-1.68; *P* < .001), age younger than 65 years (HR, 1.25; 95% CI, 1.06-1.49; *P* < .001), and age 65 years or older (HR, 1.38; 95% CI, 1.11-1.72; *P* < .001). The addition of CDK4/6 inhibitors to ET was also associated with significant PFS benefit (HR, 1.84; 95% CI, 1.70-1.98; *P* < .001) and objective response rate benefit (odds ratio, 2.02; 95% CI, 1.61-2.53; *P* < .001). However, the use of CDK4/6 inhibitors in combination with ET was associated with significantly increased risk of grade 3 or 4 adverse events compared with ET alone, including neutropenia (HR, 57.05; 95% CI, 38.26-85.05; *P* < .001), leukopenia (HR, 36.36; 95% CI, 19.35-68.34; *P* < .001), and diarrhea (HR, 4.97; 95% CI, 2.84-8.69; *P* < .001).

**Conclusions and Relevance:**

This meta-analysis indicated that, compared with ET alone, treatment with CDK4/6 inhibitors plus ET was associated with significantly improved OS, PFS, and objective response rate among patients with HR-positive, *ERBB2*-negative metastatic breast cancer.

## Key Points

**Question:**

Is the addition of cyclin-dependent kinases 4 and 6 inhibitors to endocrine therapy associated with increased overall survival vs endocrine therapy alone among patients with hormone receptor–positive, *ERBB2*-negative metastatic breast cancer?

**Findings:**

This meta-analysis of 9 randomized clinical trials with 5043 patients found that compared with endocrine therapy alone, the addition of cyclin-dependent kinases 4 and 6 inhibitors was associated with significantly increased overall survival, progression-free survival, and objective response rate among patients with hormone receptor–positive, *ERBB2*-negative metastatic breast cancer.

**Meaning:**

These results may aid physicians in selecting an effective therapeutic regimen for patients with hormone receptor–positive, *ERBB2*-negative metastatic breast cancer.

## Teaser

This systematic review and meta-analysis evaluates 9 randomized clinical trials to assess whether adding cyclin-dependent kinases 4 and 6 inhibitors to endocrine therapy is associated with increased overall survival or with adverse events vs endocrine therapy alone among patients with hormone receptor (HR)–positive, *ERBB2*-negative metastatic breast cancer.

## Introduction

Breast cancer is the most frequent malignant neoplasm observed in women and is the leading cause of cancer death.^1^ The most frequent subtype of breast cancer is hormone receptor (HR)–positive, *ERBB2* (formerly *HER2*)–negative breast cancer, which accounts for approximately 65% of all breast cancers.^2,3^ Nearly two-thirds of patients with metastatic breast cancer are HR–positive, and it is estimated that about 25% of patients with breast cancer will experience recurrence after surgery.^4,5^

Endocrine therapy (ET) is an important method of treatment for women with HR-positive, *ERBB2*-negative metastatic breast cancer. This treatment includes aromatase inhibitors, fulvestrant, tamoxifen, and others.^6^ Endocrine therapy is associated with a significant increase in both progression-free survival (PFS) and overall survival (OS).^7^ However, ET resistance and disease progression are the main causes of recurrence and death of patients.^1,6^ Ongoing alternative strategies are being explored to improve outcomes among these patients. Cyclin-dependent kinases (CDKs) regulate cell cycle progression, and CDK4 and CDK6 induce hyperphosphorylation of the retinoblastoma protein, causing the progression of tumor cells from the G1 checkpoint to the S phase of the cell cycle.^8,9,10^ The development of endocrine resistance in breast cancer is associated with the deregulation of the cyclin D/CDK4-6/retinoblastoma pathway.^11,12^ Cyclin D is also a key target for estrogen-induced cell proliferation through the estrogen receptor. Cyclin D is required for estrogen-dependent gene expression, which indicates that cyclin D expression may participate in tumorigenesis through the estrogen receptor signaling pathway, thus promoting tumor growth.^6^ A preclinical study showed that CDK4/6 inhibitors combined with ET was significantly associated with inhibiting tumor growth.^13^ The use of CDK4/6 inhibitors (ribociclib, palbociclib, and abemaciclib) is one of the most recent treatments developed for hormone receptor–positive, *ERBB2*-negative metastatic breast cancer. Although a number of studies have shown that CDK4/6 inhibitors added to ET is associated with a significant increase in PFS, OS of patients undergoing this treatment is unclear.^14,15,16,17,18,19,20,21^ Several recent randomized clinical trials have shown that treatment with CDK4/6 inhibitors and ET compared with ET alone increased OS among patients with HR-positive, *ERBB2*-negative metastatic breast cancer.^22,23,24,25,26^ However, other randomized clinical trials have found that treatment of patients with HR-positive, *ERBB2*-negative metastatic breast cancer with CDK4/6 inhibitors and ET, compared with ET alone, does not prolong OS.^4,27,28^

Thus, it remains unclear whether treatment with CDK4/6 inhibitors and ET for patients with HR-positive, *ERBB2*-negative metastatic breast cancer extends OS. Therefore, the aim of this meta-analysis is to evaluate the association between treatment with CDK4/6 inhibitors plus ET and OS.

## Methods

### Study Objectives

The primary objective was to evaluate the association of CDK4/6 inhibitors plus ET, compared with ET alone, with OS in patients with HR-positive, *ERBB2*-negative metastatic breast cancers. The secondary objective was to assess the association of PFS, objective response rate (ORR), adverse events (AEs), and grade 3 or grade 4 (grade 3/4) AEs with treatment of CDK4/6 inhibitors plus ET vs ET alone among patients with HR-positive, *ERBB2*-negative metastatic breast cancer.

### Literature Search Strategy

We conducted an electronic search of PubMed and Embase using several keywords simultaneously, including *cyclin-dependent kinases 4 and 6 inhibitor*, *palbociclib*, *ribociclib*, *abemaciclib*, *endocrine therapy*, *neoplasm*, *metastatic breast cancer*, and *advanced breast cancer*. Further searches included the main oncology conference of the European Society of Medical Oncology, the American Society of Clinical Oncology, and the San Antonio Breast Cancer Symposium databases. Only clinical trials published in English were included in the search process. Two researchers (J.L. and X.H.) independently extracted data and assessed possible bias. Searches were performed up to March 30, 2020.

### Inclusion and Exclusion Criteria

The inclusion criteria were (1) phase 2 or 3 randomized clinical trials of HR-positive, *ERBB2*-negative metastatic breast cancer (2) with patients randomly assigned to receive CDK4/6 inhibitors plus ET or ET alone, (3) having OS or PFS outcomes. The exclusion criteria were (1) phase 1 trials, (2) retrospective studies, or (3) studies without survival outcomes.

### Statistical Analysis

Data extracted from each trial included the study design, first author’s name, journal name, publication date of journal, authors’ country, patient population, menopausal status, line of therapy, phase of therapy, treatment regimens, OS, PFS, ORR, and AEs. Hazard ratios (HRs) and 95% CIs were used to evaluate PFS, OS, and subgroup analyses. Overall response and 95% CIs were used to evaluate the ORR and AEs. A random-effects model was used for analyses when *P* ≤ .10 or when the *I*^2^ statistic indicated greater than 50% study heterogeneity; otherwise, a fixed-effect model was used. Review Manager, version 5.3 (Cochrane Collaboration), was used for data analysis and forest plot production. A 2-sided *P* ≤ .01 was considered to indicate a statistically significant publication bias. This analysis was performed according to the Preferred Reporting Items for Systematic Reviews and Meta-analyses (PRISMA) reporting guideline.^29^

## Results

### Characteristics of the Included Studies

In total, 472 records were assessed in PubMed and Embase, including studies, international meeting reports, and reviews. Excluding the references, we found only 16 articles to be relevant. After application of the exclusion criteria, we determined that 9 studies were eligible for this meta-analysis (eFigure 1 in the Supplement): 1 phase 2 trial and 8 phase 3 trials reported on the efficacy and safety of treatment with CDK inhibitors plus ET vs ET alone.^16,17,22,23,25,26,28,30,31^ Thus, 9 articles, including a total of 5043 participants with HR-positive metastatic breast cancer, were included in the analysis. Relevant OS data were reported in 6 of 9 studies, and the PFS and ORR outcomes were reported in all studies. In subgroup analyses, 7 of 9 studies targeted the age of 65 years. Thus, we divided the age subgroups into patients who were younger than 65 years and patients who were 65 years or older. The main characteristics and relevant outcomes of the included studies are provided in Table 1 and in the eTable in the Supplement.^16,17,22,23,25,26,28,30,31^

**Table 1.** Main Characteristics of the Studies Included in the Meta-analysis

Source	Journal	Country	Clinical trial phase	Menopause status	Line of therapy	Drug treatment	No. of patients^a^	OS, No.^a^	PFS, No.^a^	ORR, No.^a^	Median follow-up, mo^a^
MONALEESA-7, Im et al,^25^ 2019	*The New England Journal of Medicine*	United States	3	Pre and Peri	1	Ribociclib + NSAI/TAM + GOS; NSAI/TAM + GOS	335; 337	NR; 40.9	23.8; 13.0	118; 83	34.6; 34.6
MONALEESA-2, Hortobagyi et al,^26^ 2018	*Annals of Oncology*	United States	3	Post	1	Ribociclib + letrozole; letrozole	334; 334	NR; 33.0	25.3; 16.0	142; 96	26.4; 26.4
MONALEESA-3, Slamon et al,^22^ 2020	*The New England Journal of Medicine*	United States	3	Post	1, 2	Ribociclib + fulvestrant; fulvestrant	484; 242	NR; 40.0	20.6; 12.8	157; 52	39.4; 39.4
PALOMA-1, Finn et al,^30^ 2015	*The Lancet Oncology*	United States	2	Post	1	Palbociclib + letrozole; letrozole	84; 81	37.5; 33.3	20.2; 10.2	36; 27	29.6; 27.9
PALOMA-2, Finn et al,^16^ 2016	*The New England Journal of Medicine*	United States	3	Post	1	Palbociclib + letrozole; letrozole	444; 222	NM; NM	24.8; 14.5	187; 77	23; 23
PALOMA-3, Turner et al,^23^ 2018	*The New England Journal of Medicine*	United Kingdom	3	Pre, Peri, and Post	2	Palbociclib + fulvestrant; fulvestrant	347; 174	34.9; 28.0	11.2; 4.6	36; 11	44.8; 44.8
MONARCH 2, Sledge Jr et al,^17^ 2019	*JAMA Oncology*	United States	3	Pre, Peri, and Post	1	Abemaciclib + fulvestrant; fulvestrant	446; 223	46.7; 37.3	16.4; 9.3	15; 47	47.7; 47.7
MONARCH 3, Johnston et al,^28^ 2019	*Nature Partner Journals Breast Cancer*	United Kingdom	3	Post	1	Abemaciclib + AI; AI	328; 165	NM; NM	28.2; 14.8	200; 75	26.73; 26.73
MONARCHplus, Jiang et al,^31^ 2019	*Annals of Oncology*	China	3	Post	1	Abemaciclib + NSAI; NSA; abemaciclib + fulvestrant; fulvestrant	207; 99; 104; 53^b^	NM; NM; NM; NM^b^	NR; 14.7; 11.5; 5.6^b^	115; 30; 40; 4^b^	NA; NA; NA; NA^b^

Abbreviations: AI, aromatase inhibitor; GOS, goserelin; NA, not available; NM, not mature; NR, not reached; NSAI, nonsteroidal aromatase inhibitor; ORR, objective response rate; OS, overall survival; Peri, perimenopausal; PFS, progression-free survival; Post, postmenopausal; Pre, premenopausal; TAM, tamoxifen.

^a^ Values are given for the treatment group first and the control group second.

^b^ There was more than 1 treatment group, and the control group is last.

### Overall Survival

**Figure 1.**
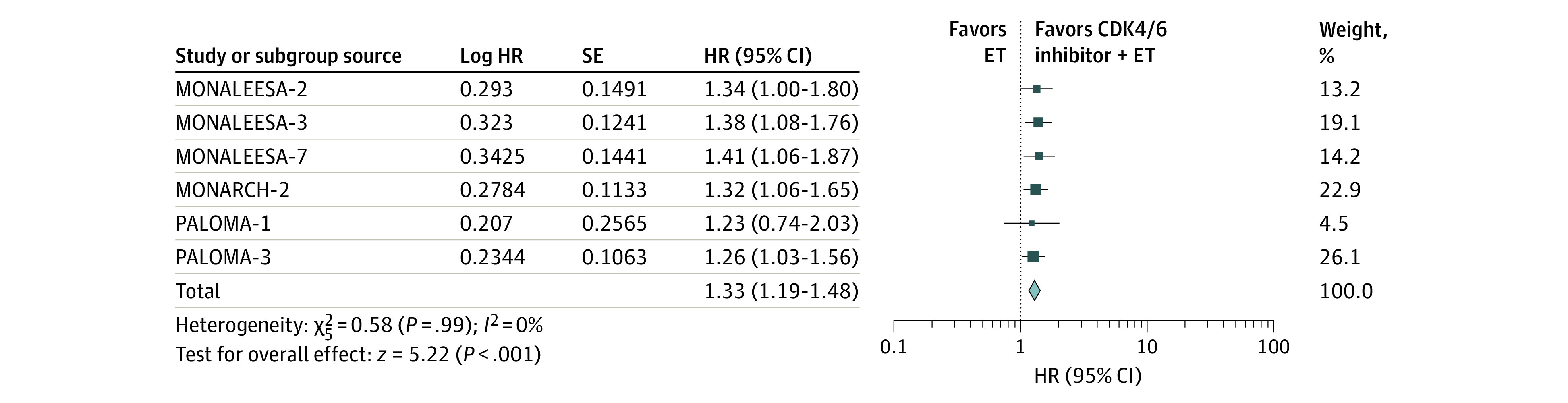
Association of Cyclin-Dependent Kinases 4 and 6 (CDK4/6) Inhibitors Plus Endocrine Therapy (ET) vs ET Alone With Overall Survival Among Women With Hormone Receptor–Positive, *ERBB2*-Negative Metastatic Breast Cancer An inverse-weighting method and a fixed-effect model were used in the analysis. The size of the data markers (squares) corresponds to the weight of the study in the meta-analysis. HR indicates hazard ratio.

In this meta-analysis, 6 studies showed indicators of OS: MONALEESA-7,^25^ MONALEESA-3,^22^ MONALEESA-2,^26^ PALOMA-1,^30^ PALOMA-3,^23^ and MONARCH 2.^17^ One phase 2 trial and 5 phase 3 trials were included to assess the association of CDK4/6 inhibitors plus ET vs ET alone with OS. For this analysis, 2030 patients were enrolled in the CDK4/6 inhibitors plus ET group and 1391 patients were enrolled in the ET group. Our results indicated that the addition of CDK4/6 inhibitors to ET was associated with significant benefit to OS (HR, 1.33; 95% CI, 1.19-1.48; *P* < .001), with low heterogeneity observed across studies *(I^2^* = 0%; *P* = .99) (Figure 1).

### Progression-Free Survival

**Figure 2.**
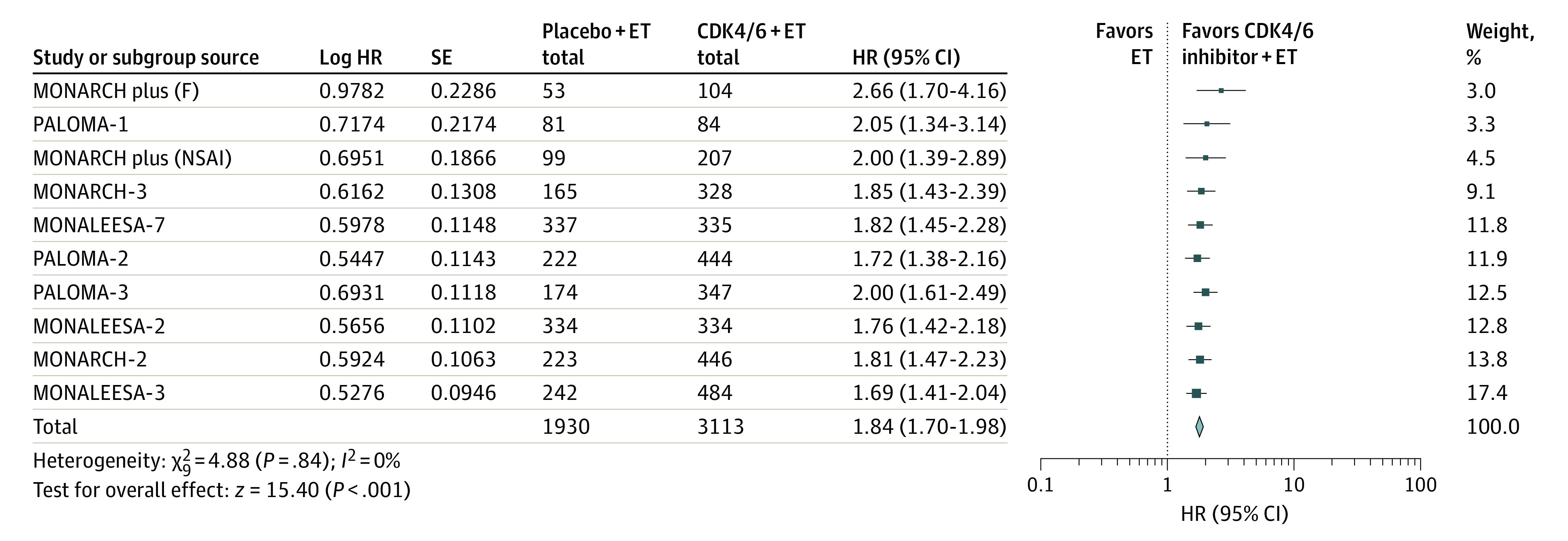
Association of Cyclin-Dependent Kinases 4 and 6 (CDK4/6) Inhibitors Plus Endocrine Therapy (ET) vs ET Alone With Progression-Free Survival Among Women With Hormone Receptor–Positive, *ERBB2*-Negative Metastatic Breast Cancer An inverse-weighting method and a random-effects model were used in the analysis. The size of the data markers (squares) corresponds to the weight of the study in the meta-analysis. F indicates fulvestrant; HR, hazard ratio; and NSAI, nonsteroidal aromatase inhibitor.

In this analysis, 1 phase 2 trial and 8 phase 3 trials were included to assess the association of CDK4/6 inhibitors plus ET vs ET alone with PFS.^16,17,22,23,25,26,28,30,31^ In these trials, 3448 patients were enrolled in the CDK4/6 inhibitors plus ET group and 2267 patients were enrolled in the ET group. Our results showed that the addition of CDK4/6 inhibitors to ET was associated with significant benefit to PFS (HR, 1.84; 95% CI, 1.70-1.98; *P* < .001), with low heterogeneity across studies (*I^2^* = 0%; *P* = .84) (Figure 2).

### Objective Response Rate

**Figure 3.**
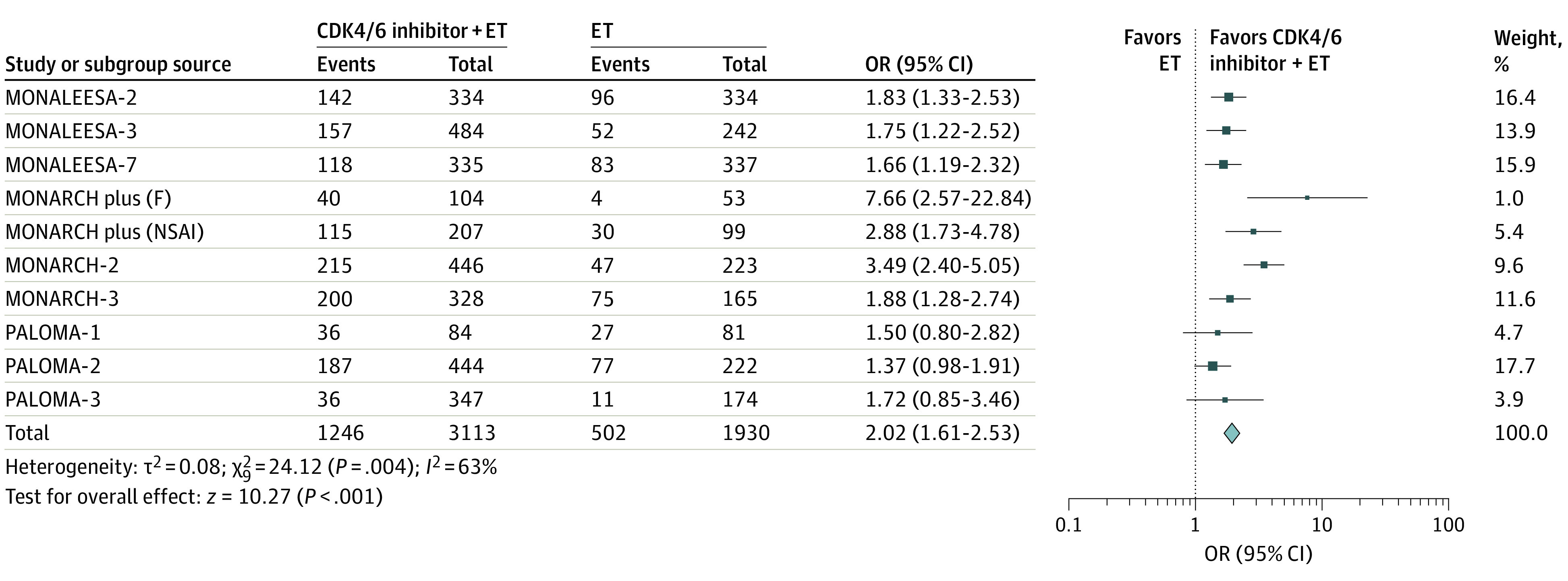
Association of Cyclin-Dependent Kinases 4 and 6 Inhibitors (CDK4/6) Plus Endocrine Therapy (ET) vs ET Alone With the Objective Response Rate Among Women With Hormone Receptor–Positive, *ERBB2*-Negative Metastatic Breast Cancer The Mantel-Haenszel method and a fixed-effect model were used in the analysis. The size of the data markers (squares) corresponds to the weight of the study in the meta-analysis. F indicates fulvestrant; OR, odds ratio; and NSAI, nonsteroidal aromatase inhibitor.

Nine studies were included to assess the association of CDK4/6 inhibitors plus ET vs ET alone with ORR.^16,17,22,23,25,26,28,30,31^ For this analysis, 3113 patients were enrolled in the CDK4/6 inhibitors plus ET group and 1930 patients were enrolled in the ET group. The addition of CDK4/6 inhibitors to ET was associated with significant benefit to ORR (odds ratio, 2.02; 95% CI, 1.61-2.53; *P* < .001), with high heterogeneity across studies (*I^2^* = 63%; *P* = .004) (Figure 3).

### OS Subgroup Analyses

#### First- and Second-Line Therapy Subgroups

For this analysis, 1436 patients were enrolled in the CDK4/6 inhibitors plus ET group and 1103 patients in the ET group of first-line therapy, whereas 584 patients were enrolled in the CDK4/6 inhibitors plus ET group and 283 patients in the ET group of second-line therapy. The combination of CDK4/6 inhibitors plus ET vs ET alone was associated with improved OS for the first-line therapy subgroup (HR, 1.35; 95% CI, 1.18-1.54; *P* < .001), with low heterogeneity across studies (*I^2^* = 0%; *P* = .99), as well as for the second-line therapy subgroup (HR, 1.30; 95% CI, 1.09-1.54; *P* < .001), also with low heterogeneity across subgroups (*I^2^* = 0%; *P* = .68) (eFigure 2 in the Supplement).

#### Premenopausal and Postmenopausal Subgroups

For this analysis, 1128 patients were enrolled in the CDK4/6 inhibitors plus ET group and 734 patients in the ET group of the premenopausal subgroup, whereas 1695 patients were enrolled in the CDK4/6 inhibitors plus ET group and 1054 patients in the ET group of the postmenopausal subgroup. Treatment with CDK4/6 inhibitors plus ET was associated with improved OS for both the premenopausal subgroup (HR, 1.32; 95% CI, 1.04-1.66; *P* < .001), with low heterogeneity across studies (*I^2^* = 0%; *P* = .41), and for the postmenopausal subgroup (HR, 1.34; 95% CI, 1.18-1.52; *P* < .001), with low heterogeneity across studies (*I^2^* = 0%; *P* = .99) (eFigure 3 in the Supplement).

#### Visceral Metastasis and Bone-Only Metastasis Subgroups

For this analysis, 2030 patients were enrolled in the CDK4/6 inhibitors plus ET group and 1391 patients in the ET group of the visceral metastasis subgroup, whereas 2030 patients were enrolled in the CDK4/6 inhibitors plus ET group and 1391 patients in the ET group of the bone-only metastasis subgroup. Treatment with CDK4/6 inhibitors plus ET was associated with improved OS both for the visceral metastasis subgroup (HR, 1.31; 95% CI, 1.12-1.53; *P* < .001), with low heterogeneity across studies (*I^2^* = 0%; *P* = .69), and for the bone-only metastasis subgroup (HR, 1.22; 95% CI, 0.88-1.68; *P* < .001), with low heterogeneity across studies (*I^2^* = 0%; *P* = .45) (eFigure 4 in the Supplement).

#### Age Subgroups

For this analysis, 1611 patients were enrolled in the CDK4/6 inhibitors plus ET group and 973 patients in the ET group of the subgroup with patients younger than 65 years, whereas 1611 patients were enrolled in the CDK4/6 inhibitors plus ET group and 973 patients in the ET group of the subgroup of patients who were 65 years of age or older. Treatment with CDK4/6 inhibitors plus ET was associated with improved OS both for the younger patient subgroup (HR, 1.25; 95% CI, 1.06-1.49; *P* < .001), with low study heterogeneity (*I^2^* = 0%; *P* = .45), and for the older patient subgroup (HR, 1.38; 95% CI, 1.11-1.72; *P* < .001), with low study heterogeneity (*I^2^* = 44%; *P* = .17) (eFigure 5 in the Supplement).

### Grade 3/4 AEs

Given that AEs are the main reason for the termination of the majority of treatment programs, we analyzed the main grade 3/4 AEs to assess their association with the treatment of patients with CDK4/6 inhibitors plus ET or ET alone. A significant increase in cases of neutropenia, leukopenia, and diarrhea was associated with treatment with CDK4/6 inhibitors plus ET. The combination of CDK4/6 inhibitors plus ET was associated with increased cases of neutropenia (HR, 57.05; 95% CI, 38.26-85.05; *P* < .001), with low study heterogeneity (*I^2^* = 46%; *P* = .07), of leukopenia (HR, 36.36; 95% CI, 19.35-68.34; *P* < .001), also with low heterogeneity (*I^2^* = 0%; *P* = .57), and of diarrhea (HR, 4.97; 95% CI, 2.84-8.69; *P* < .001), with high study heterogeneity (*I^2^* = 62%; *P* = .009) (eFigure 6 in the Supplement) (Table 2).

**Table 2.** Main Adverse Events Grade 3 or 4 Observed in the Studies Included in the Meta-analysis

Source	Drug treatment	No. of patients	No. (%) of patients with adverse event^a^					
			Neutropenia	Leukopenia	Diarrhea	Vomiting	Nausea	Fatigue
MONALEESA-7^1^	Ribociclib + NSAI/TAM + GOS	335	170 (51.0)	44 (13.0)	5 (1.0)	5 (1.0)	2 (1.0)	4 (1.0)
	NSAI/TAM + GOS	337	10 (3.0)	4 (<1.0)	1 (1.0)	2 (1.0)	1 (<1.0)	0
MONALEESA-2^13^	Ribociclib + letrozole	334	175 (52.4)	67 (20.1)	8 (2.4)	12 (3.6)	8 (2.4)	9 (2.7)
	letrozole	334	4 (1.2)	3 (0.9)	3 (0.9)	3 (0.9)	2 (0.6)	3 (0.9)
MONALEESA-3^9,32^	Ribociclib + fulvestrant	484	225 (46.6)	65 (13.5)	3 (0.6)	7 (1.4)	7 (1.4)	8 (1.7)
	Fulvestrant	242	0	0	2 (0.8)	0	2 (0.8)	1 (0.4)
PALOMA-1^17^	Palbociclib + letrozole	84	40 (48.0)	16 (19.0)	3 (4.0)	0	2 (2.0)	2 (2.0)
	Letrozole	81	1 (1.0)	0	0	1 (1.0)	1 (1.0)	1 (1.0)
PALOMA-2^25^	Palbociclib + letrozole	444	249 (56.1)	107 (24.1)	6 (1.4)	2 (0.5)	1 (0.2)	8 (1.8)
	Letrozole	222	2 (0.9)	0	3 (1.4)	3 (1.4)	4 (1.8)	1 (0.5)
PALOMA-3^10^	Palbociclib + fulvestrant	347	189 (55.0)	93 (27.0)	0	1 (<1.0)	0	8 (2.0)
	Fulvestrant	174	0	1 (1.0)	1 (1.0)	1 (1.0)	1 (1.0)	2 (1.0)
MONARCH 2^33^	Abemaciclib + fulvestrant	446	104 (23.6)	38 (8.6)	59 (13.4)	4 (0.9)	12 (2.7)	12 (2.7)
	Fulvestrant	223	3 (1.3)	0	1 (0.4)	4 (1.8)	2 (0.9)	1 (0.4)
MONARCH 3^15^	Abemaciclib + AI	328	72 (22.0)	27 (8.3)	31 (9.5)	5 (1.5)	4 (1.2)	6 (1.8)
	AI	165	1 (0.6)	0	2 (1.2)	4 (2.5)	2 (1.2)	0

Abbreviations: AI, aromatase inhibitor; GOS, goserelin; NSAI, nonsteroidal aromatase inhibitor; TAM, tamoxifen.

^a^ The denominator for each percentage is the total number of patients minus the number of deaths.

## Discussion

Endocrine therapy combined with CDK4/6 inhibitors is a reasonable option for treatment of HR-positive, *ERBB2*-negative metastatic breast cancer. Thus, we evaluated the association between CDK4/6 inhibitors combined with ET vs ET alone for treatment of patients with metastatic breast cancer and survival. There were 9 randomized clinical trials assessed, with a total of 5043 patients with metastatic breast cancer in this meta-analysis. For patients with HR-positive, *ERBB2*-negative metastatic breast cancer, treatment with ET combined with CDK4/6 inhibitors was associated not only with improved PFS and ORR but also with improved OS. Nevertheless, compared with ET alone regimens, CDK4/6 inhibitors plus ET regimens were also associated with increased risk of grade 3/4 AEs, including neutropenia, leukopenia, and diarrhea.

Two recent meta-analyses performed OS analysis, but the sample sizes in these analyses are small.^32,34^ Of these meta-analyses, one^32^ included OS data for 5 studies, whereas our meta-analysis included 6 studies that assessed OS and also analyzed subgroups of OS. Several other previously published meta-analyses and pooled analyses of PFS concluded that the use of CDK4/6 inhibitors plus ET was associated with significantly extended PFS.^2,3,13,19^ The survival data, references, and subgroup analyses included in the present meta-analysis are more comprehensive, complete, and contain a larger sample size compared with those previously published meta-analyses. Thus, the present meta-analysis has several strengths over previous analyses. Both the present meta-analysis and previous studies showed a significant benefit in OS among patients treated with CDK4/6 inhibitors plus ET in HR-positive, *ERBB2*-negative metastatic breast cancer.^22,23,24,25,26,30^ The trials using the first- or second-line setting (PALOMA-1, PALOMA-3, MONALEESA-2, MONALEESA-3, MONARCH 2, and MONALEESA-7) have published updated data prospectively showing an OS benefit with the addition of CDK4/6 inhibitors with ET.^22,23,24,25,26,30^ The PALOMA-2 and MONARCH 3 studies have not yet reported OS data because the data are not mature.^16,28^ In the present meta-analysis, 5043 patients from 9 randomized clinical trials were included to compare the treatment effects associated with the use of CDK4/6 inhibitors plus ET vs ET alone. Six of those trials showed significant benefit in OS, with low heterogeneity across the studies. In addition, 4 subgroup analyses in a previous meta-analysis^34^ also indicated improved OS associated with the combined treatment. Two of the trials concluded that the use of CDK4/6 inhibitors plus ET for HR-positive, *ERBB2*-negative metastatic breast cancer is a strong and effective treatment regimen in clinical practice.^33,35^

Our meta-analysis showed an increase in PFS among patients with HR-positive, *ERBB2*-negative metastatic breast cancer associated with the use of CDK4/6 inhibitors plus ET compared with ET alone.^16,17,22,23,25,26,28,30,31^ Our findings are consistent with the conclusions of previous meta-analyses.^36,37,38^ In addition, our results supplement and update PFS data from previous studies. Thus, previous data and the present meta-analysis suggest that treatment with CDK4/6 inhibitors plus ET vs ET is associated with extending PFS among patients with HR-positive, *ERBB2*-negative metastatic breast cancer.

Our meta-analysis showed an increase in ORR among patients with HR-positive, *ERBB2*-negative metastatic breast cancer associated with the use of CDK4/6 inhibitors plus ET compared with ET alone.^16,17,22,23,25,26,28,30,31^ We were able to conclude that the use of CDK4/6 inhibitors plus ET was associated with significantly improved ORR compared with the use of ET alone among patients with HR-positive, *ERBB2*-negative metastatic breast cancer.

Considering that various subgroups may experience different recovery results, we used subgroup analyses to assess 4 subgroups. The results of these analyses showed that, compared with the use of ET alone, treatment with CDK4/6 inhibitors plus ET was associated with significantly prolonged OS for both subgroups in the following 4 pairs of subgroups among patients with HR-positive, *ERBB2*-negative metastatic breast cancer: first-line and second-line treatment; premenopausal and postmenopausal women; visceral disease and bone-only disease; and younger than 65 years and 65 years or older. Although several previous meta-analyses have also analyzed these subgroups, they analyzed the subgroups for PFS or ORR but not for OS.^34,37^ This is an important difference between our study and those previous studies.

The addition of CDK4/6 inhibitors to treatment regimens was associated with a high risk of increased rates of neutropenia, leukopenia, and diarrhea, 3 grade 3/4 AEs that should be considered when treatment periods are long.^2^ Neutropenia, leukopenia, and diarrhea were the most common symptoms observed among patients who received CDK4/6 inhibitors plus ET treatment of metastatic breast cancer, with diarrhea being the most frequent nonhematologic grade 3/4 AE.^36,39^ However, those grade 3/4 AEs may be controlled through experience, medication, or dose adjustment. The use of the 3 CDK4/6 inhibitors was associated with different AEs: palbociclib and ribociclib with hematologic toxicities, and abemaciclib with diarrhea and fatigue.^39,40^

### Limitations

Several limitations should be acknowledged in our study. First, all the studies included in our search were in English; that is, literature in other languages on the same topic were not included. Second, all data were extracted from published literature, and no individual patient data were used in this study. The results in the meta-analysis may be biased. Third, some studies in this meta-analysis included randomized clinical trials, but the subgroup analysis did not include all of those studies. For example, in the age subgroup analysis, some studies used 40 years as the cut point, whereas other studies used 65 years as the cut point. Therefore, our results could not represent the characteristics of disease development of all patients, thus potentially affecting the interpretation of our results. However, we believe our use of carefully aggregated data and our statistical methods limited this bias. Nevertheless, our results suggested that the addition of CDK4/6 inhibitors to ET for treatment of HR-positive, *ERBB2*-negative metastatic breast cancer was associated with specific and significant benefit to OS, providing critical guidance for clinical practice.

## Conclusions

Our meta-analysis showed that, compared with ET alone, the use of CDK4/6 inhibitors plus ET was associated with significant improvements not only in PFS and ORR but also in OS among patients with HR-positive, *ERBB2*-negative metastatic breast cancer. In subgroups analyses, the addition of CDK4/6 inhibitors to ET was associated with significantly prolonged OS compared with the use of ET alone for both pairs of the following 4 patient subgroups: first-line and second-line treatment, premenopausal and postmenopausal women, visceral metastasis and bone-only metastasis, and younger than 65 years and 65 years or older. However, the addition of the CDK4/6 inhibitors to the ET regimen was associated with a higher risk of grade 3/4 AEs, especially neutropenia, leukopenia, and diarrhea. The results of this study will aid physicians in selecting an effective regimen for patients with HR-positive, *ERBB2*-negative metastatic breast cancer.

## Notes

Supplement. **eTable.** Main Characteristics of the Randomized Studies Included in the Present Meta-analysis **eFigure 1.** Search Strings and Flowcharts for Filtering and Research Selection **eFigure 2.** Effects of the Cyclin-Dependent Kinases 4 and 6 Inhibitors Plus Endocrine Therapy vs Endocrine Therapy Only on First-Line and Second-Line Subgroup Analysis of Overall Survival (OS) **eFigure 3.** Effects of the Cyclin-Dependent Kinases 4 and 6 Inhibitors Plus Endocrine Therapy vs Endocrine Therapy Only on Premenopausal and Postmenopausal Subgroup Analysis of Overall Survival (OS) **eFigure 4.** Effects of the Cyclin-Dependent Kinases 4 and 6 Inhibitors Plus Endocrine Therapy vs Endocrine Therapy Only on Visceral Metastasis and Bone Only Metastasis Subgroup Analysis of Overall Survival (OS) **eFigure 5.** Effects of the Cyclin-Dependent Kinases 4 and 6 Inhibitors Plus Endocrine Therapy vs Endocrine Therapy Only on <65 Years Old Subgroup and ≥65 Years Old Subgroup of Overall Survival (OS) **eFigure 6.** Effects of the Cyclin-Dependent Kinases 4 and 6 Inhibitors Plus Endocrine Therapy vs Endocrine Therapy Only on Grade 3/Grade 4 Adverse Events (Grade 3/4 AEs)
