# Errors in Abstract, Results, Discussion, Conclusions, and Table 1

**DOI:** 10.1001/jamanetworkopen.2020.30492

**Published:** 2020-11-19

In the Original Investigation titled “Association of Cyclin-Dependent Kinases 4 and 6 Inhibitors With Survival in Patients With Hormone Receptor–Positive Metastatic Breast Cancer: A Systematic Review and Meta-analysis,”^1^ published October 13, 2020, there was an error in the Main Outcomes and Measures section of the Abstract. The phrase given as “Overall response” should be changed to “Odds ratios.” The *P* values for second-line therapy, premenopausal women, bone-only metastasis, age younger than 65 years, and age 65 years or older in overall survival subgroup appeared as *P* < .001 but should be changed to *P* = .004 for second-line therapy, *P* = .02 for premenopausal women, *P* = .23 for bone-only metastasis, *P* = .010 for age younger than 65 years, and *P* = .003 for age 65 years or older in the Results section of the Abstract and in the Results section of the text. Also, the CDK4/6 inhibitors combined with endocrine therapy (ET) did not prolong the overall survival of patients with advanced bone-only metastasis compared with ET alone, so “bone-only metastasis (HR, 1.22; 95% CI, 0.88-1.68; *P* < .001)” should be removed from the Results section of the Abstract, “and bone-only disease” should be removed from the fifth paragraph of the Discussions section, and “bone-only metastasis” should be removed from the Conclusion section. The last 2 sentences of the seventh paragraph of the Results section should read: “Treatment with CDK4/6 inhibitors plus ET was associated with improved OS for the visceral metastasis subgroup (HR, 1.31; 95% CI, 1.12-1.53; *P* < .001), with low heterogeneity across studies (*I^2^* = 0%; *P* = .69). Treatment with CDK4/6 inhibitors plus ET was not associated with improved OS for the bone-only metastasis subgroup (HR, 1.22; 95% CI, 0.88-1.68; *P* = .23), with low heterogeneity across studies (*I^2^* = 0%; *P* = .45) (eFigure 4 in the Supplement).” Also, in Table 1, “NSA” should be changed to “NSAI” (nonsteroidal aromatase inhibitor). This article has been corrected.^1^
