# Supplementary material for: Association of Cyclin-Dependent Kinases 4 and 6 Inhibitors With Survival in Patients With Hormone Receptor–Positive Metastatic Breast Cancer: A Systematic Review and Meta-analysis
Source: JAMA Netw Open. 2020 Oct 13;3(10):e2020312. doi: 10.1001/jamanetworkopen.2020.20312 (PMC8094425; doi:10.1001/jamanetworkopen.2020.20312)
Supplement: Supplement. — eTable. Main Characteristics of the Randomized Studies Included in the Present Meta-analysis eFigure 1. Search Strings and Flowcharts for Filtering and Research Selection eFigure 2. Effects of the Cyclin-Dependent Kinases 4 and 6 Inhibitors Plus Endocrine Therapy vs Endocrine Therapy Only on First-Line and Second-Line Subgroup Analysis of Overall Survival (OS) eFigure 3. Effects of the Cyclin-Dependent Kinases 4 and 6 Inhibitors Plus Endocrine Therapy vs Endocrine Therapy Only on Premenopausal and Postmenopausal Subgroup Analysis of Overall Survival (OS) eFigure 4. Effects of the Cyclin-Dependent Kinases 4 and 6 Inhibitors Plus Endocrine Therapy vs Endocrine Therapy Only on Visceral Metastasis and Bone Only Metastasis Subgroup Analysis of Overall Survival (OS) eFigure 5. Effects of the Cyclin-Dependent Kinases 4 and 6 Inhibitors Plus Endocrine Therapy vs Endocrine Therapy Only on <65 Years Old Subgroup and ≥65 Years Old Subgroup of Overall Survival (OS) eFigure 6. Effects of the Cyclin-Dependent Kinases 4 and 6 Inhibitors Plus Endocrine Therapy vs Endocrine Therapy Only on Grade 3/Grade 4 Adverse Events (Grade 3/4 AEs) [file jamanetwopen-e2020312-s001.pdf]

Supplementary Online Content

Li J, Huo X, Zhao F, et al. Association of cyclin-dependent kinases 4 and 6 inhibitors with survival in patients with hormone receptor–positive metastatic breast cancer: a systematic review and meta-analysis. *JAMA Netw Open*. 2020;3(10):e2020312. doi:10.1001/jamanetworkopen.2020.20312

eTable. Main Characteristics of the Randomized Studies Included in the Present Meta-analysis
eFigure 1. Search Strings and Flowcharts for Filtering and Research Selection
eFigure 2. Effects of the Cyclin-Dependent Kinases 4 and 6 Inhibitors Plus Endocrine Therapy vs Endocrine Therapy Only on First-Line and Second-Line Subgroup Analysis of Overall Survival (OS)
eFigure 3. Effects of the Cyclin-Dependent Kinases 4 and 6 Inhibitors Plus Endocrine Therapy vs Endocrine Therapy Only on Premenopausal and Postmenopausal Subgroup Analysis of Overall Survival (OS)
eFigure 4. Effects of the Cyclin-Dependent Kinases 4 and 6 Inhibitors Plus Endocrine Therapy vs Endocrine Therapy Only on Visceral Metastasis and Bone Only Metastasis Subgroup Analysis of Overall Survival (OS)
eFigure 5. Effects of the Cyclin-Dependent Kinases 4 and 6 Inhibitors Plus Endocrine Therapy vs Endocrine Therapy Only on <65 Years Old Subgroup and ≥65 Years Old Subgroup of Overall Survival (OS)
eFigure 6. Effects of the Cyclin-Dependent Kinases 4 and 6 Inhibitors Plus Endocrine Therapy vs Endocrine Therapy Only on Grade 3/Grade 4 Adverse Events (Grade 3/4 AEs)

This supplementary material has been provided by the authors to give readers additional information about their work.

© 2020 Li J et al. *JAMA Network Open*.

eTable. Main Characteristics of the Randomized Studies Included in the Present Meta-analysis

Trial	Design	Population characteristics	Setting	Primary endpoint	PFS	PFS bone+	PFS viscera+	ORR
MONALEESA 7[4]	Double blind, randomized(1:1),phase III, ribociclib + tamoxifen or AI versus placebo + tamoxifen or AI	HR+ HER2−, premenopausal or perimenopausal pts, progressed during ET (adjuvant or 1°line) or DFS from adjuvant ET≤12 months	first line	PFS	HR 0.55 (95% CI 0.44–0.69)	HR 0.70 (95% CI 0.41–1.19)	HR 0.50 (95% CI 0.38–0.68)	35.1% (95% CI 30.1–40.6) in the ribociclib + tamoxifen or AI versus 24.6% (95% CI 20.2–29.6%)
MONALEESA 2[26]	Double blind, randomized(1:1), phase III trial, ribociclib + letrozole versus placebo + letrozole	HR+ HER2−, post menopausal pts, ET in neoadjuvant or adjuvant setting allowed if completed >12months	first line	PFS	HR 0.57 (95% CI 0.46–0.70)	HR 0.64 (95%CI 0.39–1.05)	HR 0.56 (CI 95% 0.42–0.74)	42.5% in the ribociclib + letrozole arm versus 28.7% in the placebo + letrozole arm
MONALEESA 3[36]	Double blind, randomized(2:1), phase III, ribociclib + fulvestrant versus placebo + fulvestrant	HR+ HER2−, post menopausal pts, newly diagnosed or relapse>12 months from neoadjuvant ET, or progressed after one line of ET	first/second line	PFS	HR 0.59 (95% CI 0.48–0.73)	HR 0.38 (95% CI 0.23–0.61)	HR 0.65 (95% CI 0.48–0.86)	32.4% (95% CI 28.3–36.6%) in the ribociclib + fulvestrant versus 21.5% (95% CI 16.3–26.7%) in placebo + fulvestrant

© 2020 Li J et al. *JAMA Network Open*.

PALOMA 1[30]	Open label, randomized, phaseII, palbociclib + letrozole versus letrozol	HR+ HER2−, post menopausal pts, ET in neoadjuvant or adjuvant setting allowed if completed >12 months	first line	PFS	HR 0.49 (95% CI 0.32–0.75)	HR 0.29 (95% CI 0.09–0.95)	HR 0.55 (95% CI 0.32–0.95)	43% (95%CI 32–54) in the palbociclib + letrozole arm versus 33% (95% CI 23–45)

PALOMA 2[16]	Double blind, randomized(2: 1), phase III, palbociclib + letrozole versus placebo + letrozole	HR+ HER2−, post menopausal pts, ET in neoadjuvant or adjuvant setting allowed if com pleted >12 months	first line	PFS	HR 0.58 (95% CI 0.46–0.72)	HR 0.36 (95% CI 0.22–0.59)	HR 0.63 (95% CI 0.47–0.85)	42.1%(95% CI 37.5–46.9) in the palbociclib + letrozole arm versus 34.7% (95% CI 28.4–41.3) in the placebo + letrozole arm
PALOMA 3[37]	Double blind, randomized(2: 1), phase III, Palbociclib + Fulvestrant versus placebo + fulvestr-ant	HR+ HER2−, post menopausal pts or pre-peri menopausal, pts progressed during ET (adju vant or 1°line) or DFS from adjuvant ET≤12 months	second line	PFS	HR 0.46 (95% CI 0.36–0.59)	HR 0.36 (95% CI 0.22–0.60)	HR 0.47 (95% CI 0.34–0.63)	10.4% (95% CI 7.4–14.1) in the palbociclib + ful vestrant arm versus 6.3% (95% CI 3.2–11.0) in the placebo + fulvestrant arm

© 2020 Li J et al. *JAMA Network Open*.

MONARCH 2[39]	Double blind, randomized (2:1), phase III, abemaciclib + fulvestrant versus placebo + fulvestrant	HR+ HER2−, post menopausal pts or pre-peri menopausal, pts progressed during ET (adjuvant or 1°line) or DFS from adjuvant ET≤12 months	second line	PFS	HR 0.55 (95% CI 0.45–0.68)	HR 0.54 (95% CI 0.36–0.83)	HR 0.48 (95% CI 0.37–0.63)	48.1% (95% CI 42.8%–53.6%) in the abemaciclib + fulvestrant arm versus 21.3% (95% CI 27.3%–41.8%)in the placebo + fulvestrant arm
MONARCH 3[14]	Double blind, randomized(2:1), phase III, abemaciclib + AI (letrozole or anastrozole) versus abemaciclib + AI	HR+ HER2−, post menopausal pts, ET in neoadjuvant or adjuvant setting allowed if com pleted >12 months	first line	PFS	HR 0.54 (95% CI 0.42–0.70)	HR 0.57 (95%CI 0.31–1.04)	HR 0.57 (95% CI 0.41–0.79)	61.0% in the abemaciclib+AI arm versus 45.5% in the placebo+AI arm
MONARCH plus[31]	double-blind, randomized(2:1), phase III, Abemaciclib + NSAI versus placebo + NSAI	HR+ HER2−, post menopausal pts,	first line	PFS	HR 0.50 (95% CI 0.35–0.72)	Not available	Not available	56% in the abemaciclib + NSAI arm versus 30% in the placebo + NSAI arm
	double-blind, randomized(2:1), phase III, Abemaciclib +	HR+ HER2−, post menopausal pts,	first line	PFS	HR 0.38 (95% CI	Not available	Not available	39% in the abemaciclib + fulvestrant arm versus 8% in the placebo + fulvestrant arm

© 2020 Li J et al. *JAMA Network Open*.

	fulvestrant versus placebo + fulvestrant				0.24–0.59)			

ET: endocrine therapy, HR+: hormone receptor positive, HER2−: human epidermal growth factor receptor 2 negative, ORR: overall response rates, PFS : progression-free survival, pts: patients, HR: hazard ratios, CI: confidence intervals, AI: aromatase inhibitor, NSAI: nonsteroidal aromatase inhibitor.

© 2020 Li J et al. *JAMA Network Open*.

eFigure 1. Search Strings and Flowcharts for Filtering and Research Selection

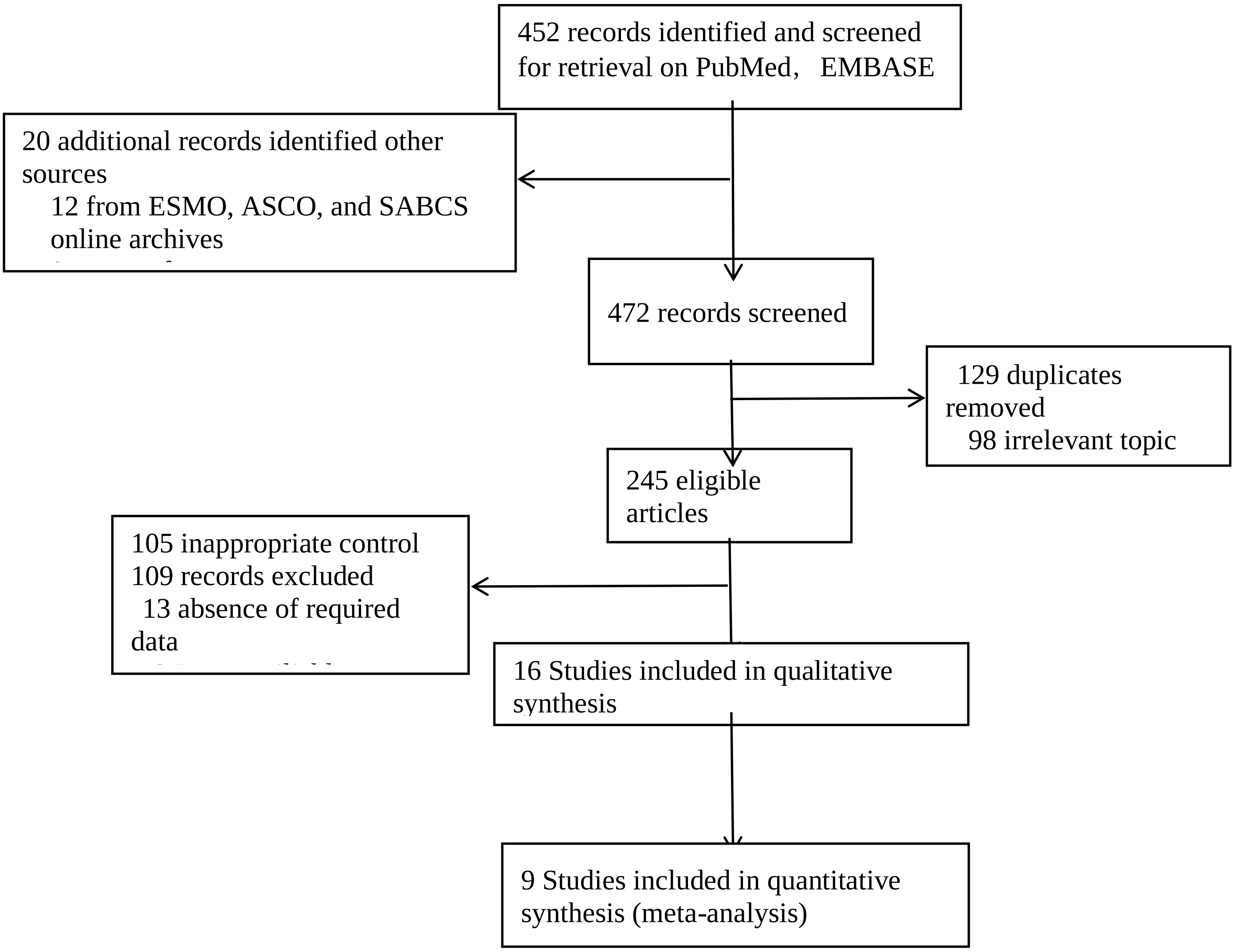

© 2020 Li J et al. *JAMA Network Open*.

eFigure 2. Effects of the Cyclin-Dependent Kinases 4 and 6 Inhibitors Plus Endocrine Therapy vs Endocrine Therapy Only on First-Line and Second-Line Subgroup Analysis of Overall Survival (OS)

Study or Subgroup	log[Hazard Ratio]	SE	Weight	Hazard Ratio IV, Fixed, 95% CI
**1.2.1 1 Line**				
MONALEESA-2	0.293	0.1491	13.2%	1.34 [1.00, 1.80]
MONALEESA-3	0.3567	0.1923	8.0%	1.43 [0.98, 2.08]
MONALEESA-7	0.3425	0.1441	14.2%	1.41 [1.06, 1.87]
MONARCH-2	0.2784	0.1133	22.9%	1.32 [1.06, 1.65]
PALOMA-1	0.207	0.2566	4.5%	1.23 [0.74, 2.03]
**Subtotal (95% CI)**			**62.8%**	**1.35 [1.18, 1.54]**
Heterogeneity: Chi² = 0.34, df = 4 (P = 0.99); I² = 0%				
Test for overall effect: Z = 4.39 (P < 0.0001)				
**1.2.2 2 Line**				
MONALEESA-3	0.3147	0.162	11.2%	1.37 [1.00, 1.88]
PALOMA-3	0.2344	0.1063	26.0%	1.26 [1.03, 1.56]
**Subtotal (95% CI)**			**37.2%**	**1.30 [1.09, 1.54]**
Heterogeneity: Chi² = 0.17, df = 1 (P = 0.68); I² = 0%				
Test for overall effect: Z = 2.91 (P = 0.004)				
**Total (95% CI)**			**100.0%**	**1.33 [1.20, 1.48]**
Heterogeneity: Chi² = 0.66, df = 6 (P = 1.00); I² = 0%				
Test for overall effect: Z = 5.26 (P < 0.00001)				
Test for subgroup differences: Chi² = 0.14, df = 1 (P = 0.71), I² = 0%				

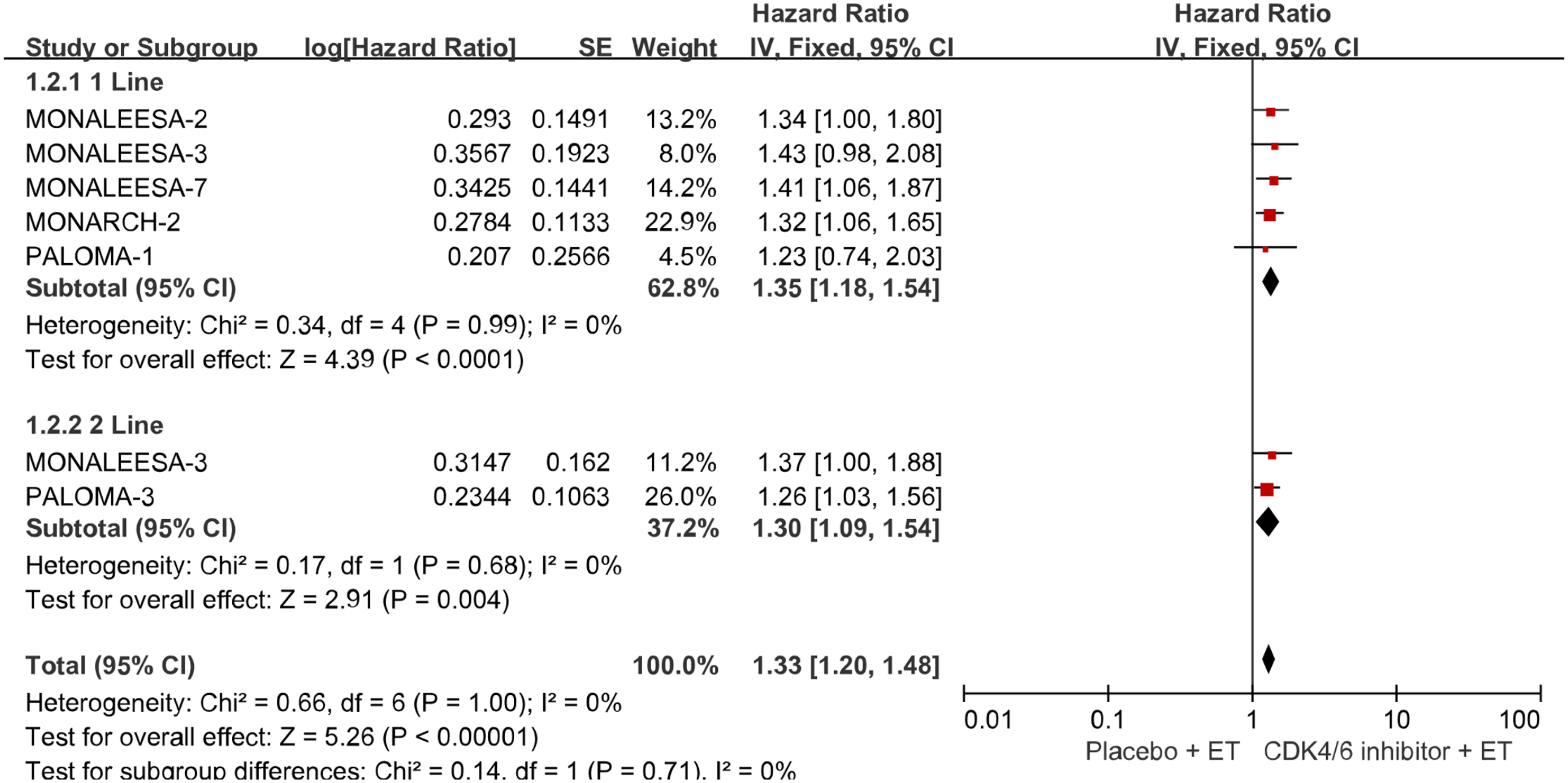

© 2020 Li J et al. *JAMA Network Open*.

eFigure 3. Effects of the Cyclin-Dependent Kinases 4 and 6 Inhibitors Plus Endocrine Therapy vs Endocrine Therapy Only on Premenopausal and Postmenopausal Subgroup Analysis of Overall Survival (OS)

Study or Subgroup	log[Hazard Ratio]	SE	Weight	Hazard Ratio IV, Fixed, 95% CI
**1.3.1 premenopausalc**				
MONALEESA-7	0.3425	0.1441	15.6%	1.41 [1.06, 1.87]
MONARCH-2	0.3725	0.3048	3.5%	1.45 [0.80, 2.64]
PALOMA-3	-0.0676	0.2844	4.0%	0.93 [0.54, 1.63]
**Subtotal (95% CI)**			**23.1%**	**1.32 [1.04, 1.66]**
Heterogeneity: Chi² = 1.77, df = 2 (P = 0.41); I² = 0%				
Test for overall effect: Z = 2.33 (P = 0.02)				
**1.3.2 postmenopausalc**				
MONALEESA-2	0.293	0.1875	9.2%	1.34 [0.93, 1.94]
MONALEESA-3	0.3285	0.1221	21.7%	1.39 [1.09, 1.76]
MONARCH-2	0.2575	0.1214	22.0%	1.29 [1.02, 1.64]
PALOMA-1	0.207	0.2566	4.9%	1.23 [0.74, 2.03]
PALOMA-3	0.3147	0.1303	19.1%	1.37 [1.06, 1.77]
**Subtotal (95% CI)**			**76.9%**	**1.34 [1.18, 1.52]**
Heterogeneity: Chi² = 0.31, df = 4 (P = 0.99); I² = 0%				
Test for overall effect: Z = 4.51 (P < 0.00001)				
**Total (95% CI)**			**100.0%**	**1.33 [1.19, 1.49]**
Heterogeneity: Chi² = 2.10, df = 7 (P = 0.95); I² = 0%				
Test for overall effect: Z = 5.08 (P < 0.00001)				
Test for subgroup differences: Chi² = 0.02, df = 1 (P = 0.90), I² = 0%				

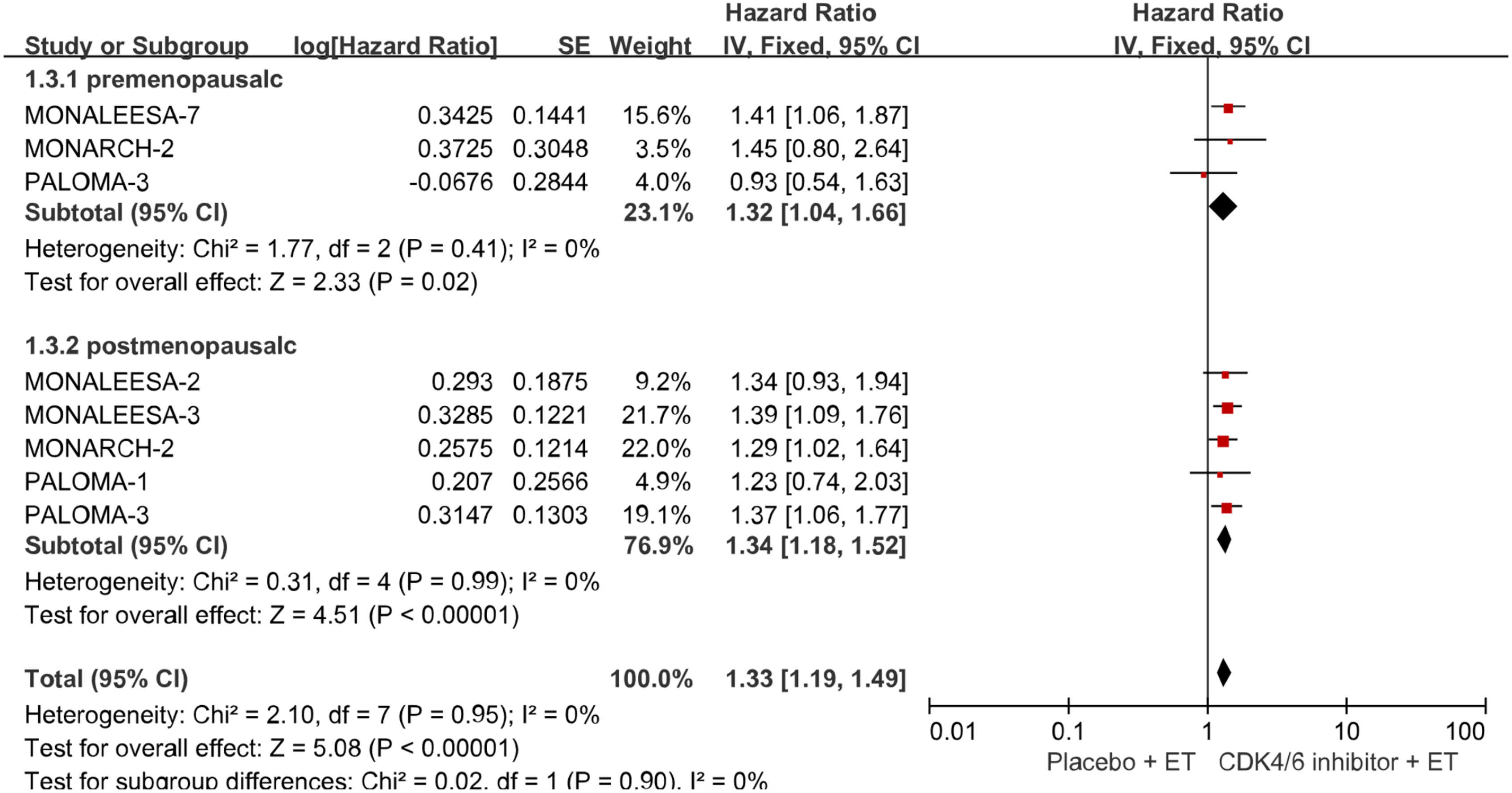

© 2020 Li J et al. *JAMA Network Open*.

eFigure 4. Effects of the Cyclin-Dependent Kinases 4 and 6 Inhibitors Plus Endocrine Therapy vs Endocrine Therapy Only on Visceral Metastasis and Bone Only Metastasis Subgroup Analysis of Overall Survival (OS)

Study or Subgroup	log[Hazard Ratio]	SE	Weight	Hazard Ratio IV, Fixed, 95% CI
**1.4.1 viscus**				
MONALEESA-3	0.2107	0.1679	17.9%	1.23 [0.89, 1.72]
MONALEESA-7	0.3147	0.1893	14.1%	1.37 [0.95, 1.99]
MONARCH-2	0.3931	0.1418	25.1%	1.48 [1.12, 1.96]
PALOMA-3	0.1625	0.145	24.0%	1.18 [0.89, 1.56]
**Subtotal (95% CI)**			**81.1%**	**1.31 [1.12, 1.53]**
Heterogeneity: Chi² = 1.48, df = 3 (P = 0.69); I² = 0%				
Test for overall effect: Z = 3.43 (P = 0.0006)				
**1.4.2 bone**				
MONALEESA-3	0.5108	0.3001	5.6%	1.67 [0.93, 3.00]
MONALEESA-7	0	0.3297	4.6%	1.00 [0.52, 1.91]
MONARCH-2	0.0976	0.2421	8.6%	1.10 [0.69, 1.77]
**Subtotal (95% CI)**			**18.9%**	**1.22 [0.88, 1.68]**
Heterogeneity: Chi² = 1.62, df = 2 (P = 0.45); I² = 0%				
Test for overall effect: Z = 1.20 (P = 0.23)				
**Total (95% CI)**			**100.0%**	**1.29 [1.12, 1.49]**
Heterogeneity: Chi² = 3.27, df = 6 (P = 0.77); I² = 0%				
Test for overall effect: Z = 3.61 (P = 0.0003)				
Test for subgroup differences: Chi² = 0.17, df = 1 (P = 0.68), I² = 0%				

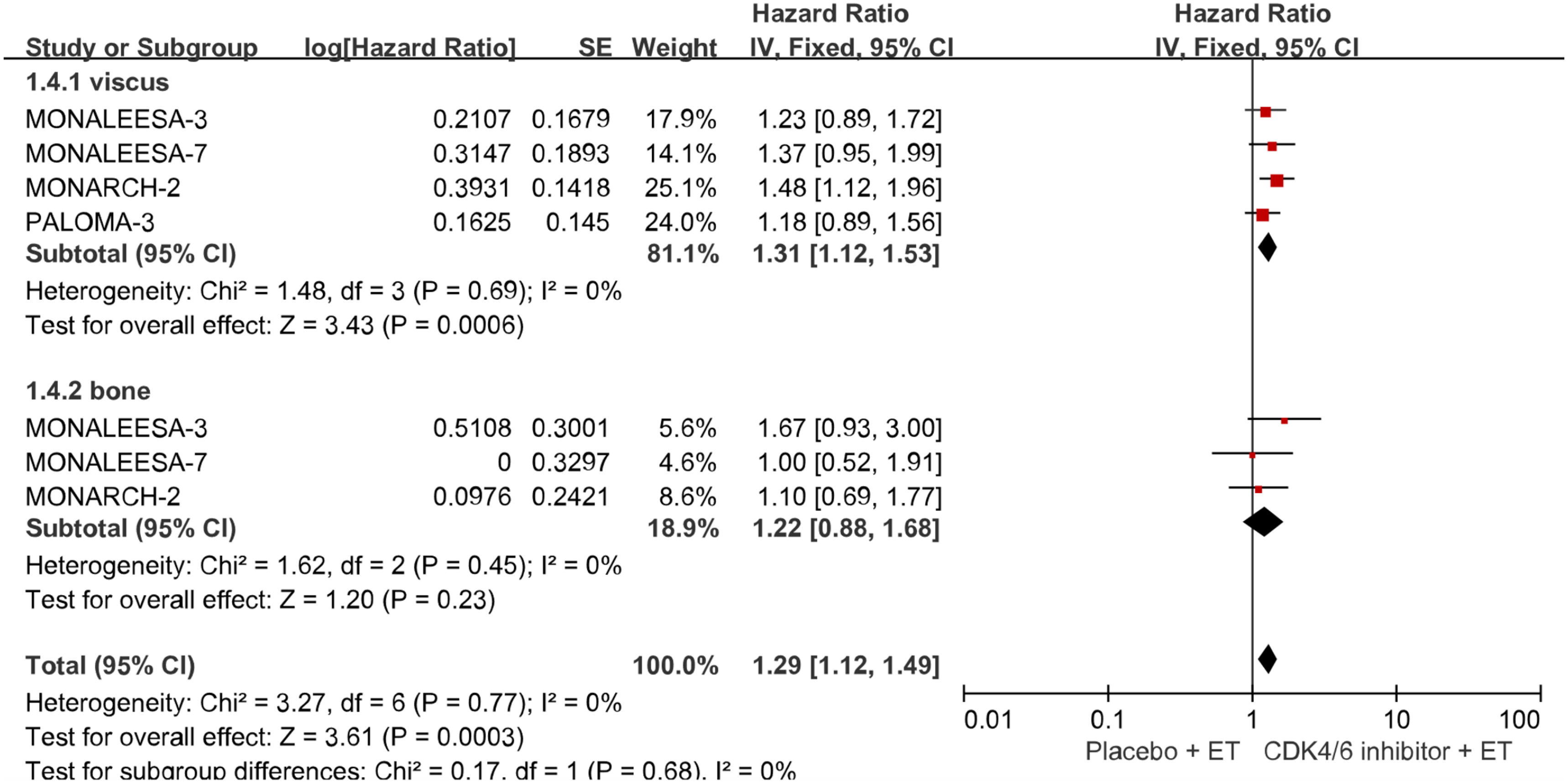

© 2020 Li J et al. *JAMA Network Open*.

eFigure 5. Effects of the Cyclin-Dependent Kinases 4 and 6 Inhibitors Plus Endocrine Therapy vs Endocrine Therapy Only on <65 Years Old Subgroup and ≥65 Years Old Subgroup of Overall Survival (OS)

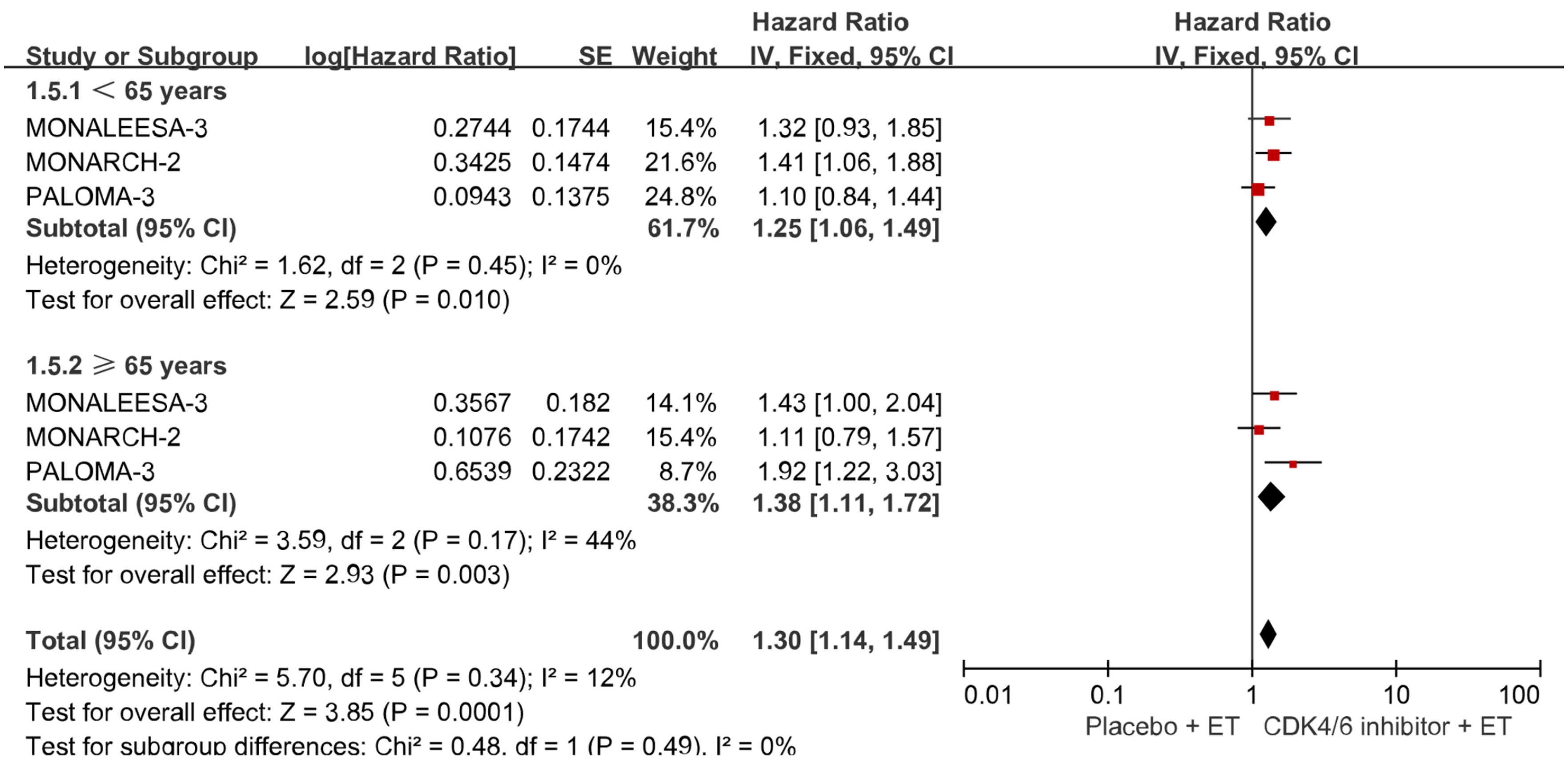

© 2020 Li J et al. *JAMA Network Open*.

eFigure 6. Effects of the Cyclin-Dependent Kinases 4 and 6 Inhibitors Plus Endocrine Therapy vs Endocrine Therapy Only on Grade 3/Grade 4 Adverse Events (Grade 3/4 AEs)

Study or Subgroup	Experimental Events	Experimental Total	Control Events	Control Total	Weight	Odds Ratio M-H, Fixed, 95% CI
**1.10.1 Neutropenia**						
MONALEESA-2	170	334	10	334	5.1%	33.59 [17.27, 65.30]
MONALEESA-3	225	484	0	242	0.4%	421.45 [26.14, 6796.12]
MONALEESA-7	171	335	10	337	5.1%	34.10 [17.54, 66.28]
MONARCH-2	104	446	3	223	3.2%	22.30 [6.99, 71.15]
MONARCH-3	72	328	1	165	1.1%	46.13 [6.35, 335.16]
PALOMA-1	40	84	1	81	0.6%	72.73 [9.67, 547.21]
PALOMA-2	249	444	2	222	1.2%	140.46 [34.47, 572.31]
PALOMA-3	189	347	0	174	0.3%	417.26 [25.79, 6751.28]
**Subtotal (95% CI)**		**2802**		**1778**	**17.0%**	**57.05 [38.26, 85.05]**
Total events	1220		27			
Heterogeneity: Chi² = 12.89, df = 7 (P = 0.07); I² = 46%						
Test for overall effect: Z = 19.85 (P < 0.00001)						
**1.10.2 Leukopenia**						
MONALEESA-2	67	334	3	334	2.5%	27.69 [8.61, 89.01]
MONALEESA-3	65	484	0	242	0.6%	75.73 [4.67, 1229.04]
MONALEESA-7	44	335	4	337	3.6%	12.59 [4.47, 35.46]
MONARCH-2	38	446	0	223	0.6%	42.13 [2.58, 689.00]
MONARCH-3	27	328	0	165	0.6%	30.19 [1.83, 498.12]
PALOMA-1	16	84	0	81	0.4%	39.26 [2.31, 666.51]
PALOMA-2	107	444	0	222	0.5%	141.74 [8.76, 2292.50]
PALOMA-3	93	347	1	174	1.0%	63.34 [8.75, 458.74]
**Subtotal (95% CI)**		**2802**		**1778**	**10.0%**	**36.36 [19.35, 68.34]**
Total events	457		8			
Heterogeneity: Chi² = 5.76, df = 7 (P = 0.57); I² = 0%						
Test for overall effect: Z = 11.16 (P < 0.00001)						
**1.10.3 Diarrhea**						
MONALEESA-2	8	334	3	334	3.1%	2.71 [0.71, 10.30]
MONALEESA-3	3	484	2	242	2.8%	0.75 [0.12, 4.51]
MONALEESA-7	5	335	1	337	1.0%	5.09 [0.59, 43.81]
MONARCH-2	59	446	1	223	1.2%	33.84 [4.66, 245.96]
MONARCH-3	31	328	2	165	2.5%	8.51 [2.01, 36.00]
PALOMA-1	3	84	0	81	0.5%	7.00 [0.36, 137.68]
PALOMA-2	6	444	3	222	4.1%	1.00 [0.25, 4.04]
PALOMA-3	0	347	1	174	2.1%	0.17 [0.01, 4.11]
**Subtotal (95% CI)**		**2802**		**1778**	**17.3%**	**4.97 [2.84, 8.69]**
Total events	115		13			
Heterogeneity: Chi² = 18.62, df = 7 (P = 0.009); I² = 62%						
Test for overall effect: Z = 5.62 (P < 0.00001)						
**1.10.4 Nausea**						
MONALEESA-2	8	334	2	334	2.0%	4.07 [0.86, 19.33]
MONALEESA-3	7	484	2	242	2.7%	1.76 [0.36, 8.54]
MONALEESA-7	2	335	1	337	1.0%	2.02 [0.18, 22.36]
MONARCH-2	12	446	2	223	2.7%	3.06 [0.68, 13.77]
MONARCH-3	4	328	2	165	2.7%	1.01 [0.18, 5.55]
PALOMA-1	2	84	1	81	1.0%	1.95 [0.17, 21.95]
PALOMA-2	1	444	4	222	5.5%	0.12 [0.01, 1.11]
PALOMA-3	0	347	1	174	2.1%	0.17 [0.01, 4.11]
**Subtotal (95% CI)**		**2802**		**1778**	**19.9%**	**1.47 [0.81, 2.66]**
Total events	36		15			
Heterogeneity: Chi² = 9.58, df = 7 (P = 0.21); I² = 27%						
Test for overall effect: Z = 1.27 (P = 0.20)						
**1.10.5 Vomiting**						
MONALEESA-2	12	334	3	334	3.0%	4.11 [1.15, 14.71]
MONALEESA-3	7	484	0	242	0.7%	7.62 [0.43, 133.94]
MONALEESA-7	5	335	2	337	2.0%	2.54 [0.49, 13.17]
MONARCH-2	4	446	4	223	5.5%	0.50 [0.12, 2.00]
MONARCH-3	5	328	4	165	5.5%	0.62 [0.17, 2.35]
PALOMA-1	0	84	1	81	1.6%	0.32 [0.01, 7.91]
PALOMA-2	2	444	3	222	4.2%	0.33 [0.05, 1.99]
PALOMA-3	1	347	1	174	1.4%	0.50 [0.03, 8.04]
**Subtotal (95% CI)**		**2802**		**1778**	**23.8%**	**1.32 [0.76, 2.29]**
Total events	36		18			
Heterogeneity: Chi² = 11.73, df = 7 (P = 0.11); I² = 40%						
Test for overall effect: Z = 0.99 (P = 0.32)						
**1.10.6 Fatigue**						
MONALEESA-2	9	334	3	334	3.0%	3.06 [0.82, 11.39]
MONALEESA-3	8	484	1	242	1.4%	4.05 [0.50, 32.57]
MONALEESA-7	4	335	0	337	0.5%	9.16 [0.49, 170.86]
MONARCH-2	12	446	1	223	1.4%	6.14 [0.79, 47.51]
MONARCH-3	6	328	0	165	0.7%	6.67 [0.37, 119.15]
PALOMA-1	2	84	1	81	1.0%	1.95 [0.17, 21.95]
PALOMA-2	8	444	1	222	1.4%	4.06 [0.50, 32.63]
PALOMA-3	8	347	2	174	2.7%	2.03 [0.43, 9.66]
**Subtotal (95% CI)**		**2802**		**1778**	**12.1%**	**3.76 [1.90, 7.45]**
Total events	57		9			
Heterogeneity: Chi² = 1.72, df = 7 (P = 0.97); I² = 0%						
Test for overall effect: Z = 3.80 (P = 0.0001)						
**Total (95% CI)**		**16812**		**10668**	**100.0%**	**15.21 [12.43, 18.61]**
Total events	1921		90			
Heterogeneity: Chi² = 243.31, df = 47 (P < 0.00001); I² = 81%						
Test for overall effect: Z = 26.46 (P < 0.00001)						
Test for subgroup differences: Chi² = 194.12, df = 5 (P < 0.00001), I² = 97.4%						

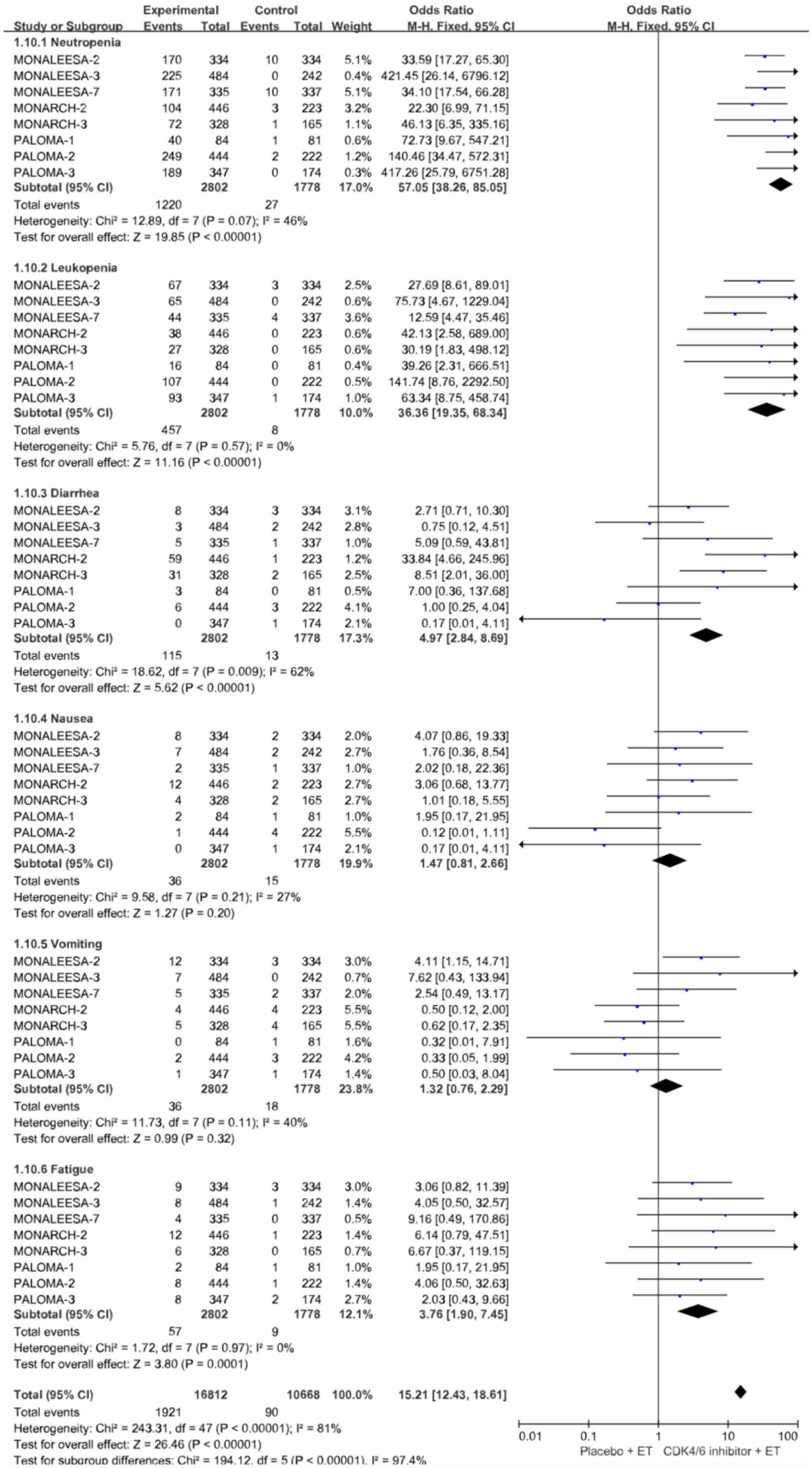

© 2020 Li J et al. *JAMA Network Open*.
